# Mediterranean Diet Affects Blood Circulating Lipid-Soluble Micronutrients and Inflammatory Biomarkers in a Cohort of Breast Cancer Survivors: Results from the SETA Study

**DOI:** 10.3390/nu13103482

**Published:** 2021-09-30

**Authors:** Mara Negrati, Claudia Razza, Claudia Biasini, Camilla Di Nunzio, Alessandra Vancini, Margherita Dall’Asta, Giorgia Lovotti, Erminio Trevisi, Filippo Rossi, Luigi Cavanna

**Affiliations:** 1 Clinical Nutrition Unit, Medicine Department, “G. da Saliceto” Hospital, Via Taverna 49, 29121 Piacenza, Italy; m.negrati2@ausl.pc.it (M.N.); claudia.razza@gmail.com (C.R.); vancini.alessandra@libero.it (A.V.); 2 Oncology Unit, Onco-Hematology Department, “G. da Saliceto” Hospital, Via Taverna 49, 29121 Piacenza, Italy; c.biasini@ausl.pc.it (C.B.); c.dinunzio@ausl.pc.it (C.D.N.); 3 Department of Animal Science, Food and Nutrition, Università Cattolica del Sacro Cuore, 29122 Piacenza, Italy; margherita.dallasta@unicatt.it (M.D.); giorgia.lovotti@unicatt.it (G.L.); erminio.trevisi@unicatt.it (E.T.); l.cavanna@ausl.pc.it (L.C.)

**Keywords:** breast cancer recurrence, secondary prevention, carotenoids, Mediterranean diet, inflammation

## Abstract

Breast cancer (BC) is a major public health concern and substantial research has shown that adhering to a healthy dietary pattern, such as the Mediterranean Diet (MD), may prevent the onset of cancer and BC relapses. This study aims at specifically investigating the association of MD with circulating dietary-related biomarkers in a cohort of BC survivors. Eighty patients (mean age of 54.9 ± 10.6) with a histologically confirmed diagnosis of BC who had not received any pharmacological or radiotherapy treatment for at least two months were enrolled. Fasting serum lipid-soluble vitamins (retinol, tocopherol), plant pigments (β-carotene, lutein + zeaxanthin, cryptoxanthin, lycopene), inflammatory and oxidative stress markers (ceruloplasmin; haptoglobin; paraoxonases; reactive oxygen molecule; thiol groups, Ferric reducing antioxidant power), and cardiometabolic parameters (body mass index (BMI); glucose; insulin; HOMA-IR; total cholesterol; LDL-cholesterol; HDL-cholesterol; triglycerides) were analyzed. Adherence to the MD was assessed through the Mediterranean Diet Score (MDS) questionnaire. Fasting blood samples were collected for the evaluation of selected biomarkers. MDS resulted positively correlated with β-carotene (r 0.331; *p* < 0.01) and lycopene (r 0.274; *p* < 0.05) and negatively with retinol (r −0.346; *p* < 0.05). Among the investigated inflammatory biomarkers, MDS was only correlated with antioxidant capacity (r 0.256; *p* < 0.05), while none of the investigated cardiometabolic parameters were significantly correlated with this index. The strong significant correlation between β-carotene and MDS encourages us to consider this pro-vitamin as a putative biomarker to take into account for evaluating the adherence to the MD.

## 1. Introduction

Breast cancer (BC) is the most common cancer affecting women worldwide, accounting for about 2.09 million cases and 627,000 deaths in 2018 (https://www.who.int/en/news-room/fact-sheets/detail/cancer (accessed on 14 April 2021)). Modifiable lifestyle-based risk factors such as tobacco use [1], alcohol consumption, bodyweight and unhealthy diet (e.g., low intake of vegetables and fiber, high intake of red meat, processed meat, and deep-frying fat) [2,3,4], and physical inactivity [5] have been recognized to play a key role in the onset of BC [6].

A large body of evidence has recognized the Mediterranean Diet (MD) as a dietary regimen that helps promote longevity [7], prevent chronic diseases [8] such as type 2 diabetes [9], cardiovascular diseases [10], and cancer [3,10,11,12]. The relationship between diet and BC has been widely investigated [13], and several reports clearly indicate that certain dietary patterns may prevent the occurrence of BC. The MD, which is mainly based on the consumption of fruit and vegetables, wholegrain cereals, and legumes, seems to be one of the most effective in reducing the incidence of cancer [10]. The beneficial effect of the MD on BC prevention might be attributed to the indirect effect of specific nutrients or bioactive compounds on BC, inflammation, DNA damage and repair, oxidative stress, and genetic modifications [14,15]. Although there is a large body of evidence supporting the role of lifestyle factors on the incidence of BC, there are still only a few studies that clearly support their role in the survival rates for BC [6].

Women who have been treated for BC present an increased risk of both primary cancer relapse and weight gain and develop comorbidities or secondary cancers [16,17]. Furthermore, their quality of life is reduced as a result of the treatment for the primary BC [18]. For this reason, cancer survivors are advised to follow a healthy diet, achieve and maintain a healthy weight, and take regular exercise [5,6,19].

In the past, attempts were made to create suitable tools for the assessment of adherence to the MD. Several diet quality indices have been proposed [20,21,22,23,24]. Of these, some were specifically created in order to investigate the adherence to the MD of patients with cancer, which differs from others because alcohol consumption was negatively scored due to its role in cancer promotion. In recent years, several studies have investigated the association of indices of adherence to the MD with circulating serum molecules in various population groups in order to best understand which biomarkers were most representative of this type of dietary pattern, although only a few of the research works were conducted on BC survivors.

Therefore, the present research was aimed to verify the hypothesis that MDS may affect blood levels of lipid-soluble vitamins, carotenoids, and inflammatory and anthropometric parameters in a cohort of BC survivors from the SETA study.

## 2. Materials and Methods

### 2.1. Study Design

This was a cross-sectional observational study comprising a cohort of BC survivors within the SETA (SEnologia e Terapia Alimentare) study. The study started in 2012 at the Oncology-Hematology Department of Piacenza Hospital (Italy) and participants were recruited until 2014. The protocol was approved by the local Ethics Committee (approval number: 840), and the study was conducted in accordance with the Helsinki Declaration. Prior to the study starting, participants were informed about the aims and procedures of the study and provided their written informed consent. The volunteers were women with a histologically confirmed diagnosis of BC, who had not received any pharmacological or radiotherapy treatment for at least two months. The exclusion criteria of the study were: metastatic BC; pregnancy or breastfeeding; illness and pathological, social, psychological, or family conditions that did not allow adherence to the treatments or made compliance with the procedures of the research difficult; and the presence of any other malignant tumors. The eligible patients enrolled were assessed for MDS evaluation, anthropometric parameters, and blood samples were collected for biomarker analysis.

### 2.2. Dietary Assessment

The questionnaire formulated by Demetriou et al. [24] was administered to each patient in order to verify adherence to the MD. This score is based on the frequency of consumption of the food groups present in the MD pyramid. Items recommended for consumption are scored positively (0–5 points): (1) all cereals (bread, pasta, rice, etc.); (2) potatoes; (3) fruit; (4) vegetables; (5) legumes; (6) fish; (7) olive oil. Those that are recommended infrequently (i.e., never/once a month), are reverse scored based on consumption (5–0 points): (8) red meat products; (9) poultry; (10) full-fat dairy products (cheese, yogurt, milk). Alcohol (ml/day) is scored 5 points attributed to an intake of 0 mL up to 0 for a dose >700 mL. The maximum score is 55.

### 2.3. Anthropometric Parameters

Bodyweight (BW), height (H), and body mass index (BMI) were measured using a calibrated scale with a stadiometer (GIMA). The volunteers were asked not to exercise or consume any food or drink two hours before measurements were taken, and they were wearing only underwear. BMI (kg/m^2^) was calculated for every patient based on weight and height. Waist circumference was also measured, using a constant tension tape positioned under the midline of the participant’s armpit, at the midpoint between the lower part of the last rib and the top of the hip, at the end of a normal expiration with the arms relaxed at the sides.

### 2.4. Biomarker Analysis

Fasting blood samples were collected into heparinized tubes and the plasma was centrifuged and stored at −80 °C until analysis. A clinical autoanalyzer (ILAB-650, Instrumentation Laboratory, Bedford, MA, USA) was used to determine the concentration of aspartate amino transferase–glutamate oxaloacetate transaminase (AST-GOT), γ-glutamyl transferase (GGT), aspartate amino transferase-glutamic pyruvate transaminase (ALT/GPT), total protein, albumin, haptoglobin, ceruloplasmin, reactive oxygen metabolites (ROMs), ferric-reducing antioxidant power (FRAP), paraoxonase (PON) and thiol groups. Globulin was calculated as the difference between total protein and albumin. AST-GOT, GGT, ALT/GPT, total protein, and albumin were determined by means of a kit purchased from IL (Instrumentation Laboratory spa, Bedford, MA, USA).

The ROMs and thiol groups were determined with a kit purchased from Diacron International srl, adapted to ILAB 650. The FRAP was determined following the method proposed by Jacometo et al. [25]. The haptoglobin level in the blood was determined by the methods proposed by Skinner et al. (1991) [26]; ceruloplasmin was determined with the method proposed by Sunderman and Nomoto (1970) [27]. Paraoxonase was determined by the method of Ferré et al. (2002) [28], adapted to the ILAB 650, as previously described by Bionaz et al. [29].

Glucose, insulin, and blood lipids (total cholesterol, LDL-cholesterol, HDL-cholesterol, triglycerides) were analyzed by the internal laboratory of the hospital. The homeostatic model assessment of insulin resistance (HOMA-IR) was then derived [30]. Concentrations of retinol, tocopherol, β-carotene, lycopene, β-cryptoxanthin, zeaxanthin, and lutein were analyzed by reversed-phase HPLC (LC-4000, Jasco Europe SRL, Cremella, Italy). Plasma vitamins and pigments were extracted with n-hexane and analyzed by reverse-phase HPLC using ZORBAX Eclipse Plus C18, 4.6 × 150 mm, 3.5 µ column (Agilent Technologies Inc, Santa Clara, CA, USA); a UV-VIS detector set at 325 nm (for retinol), 290 nm (for tocopherol), 450 nm (for β-carotene, lycopene, β-cryptoxanthin, zeaxanthin, and lutein); and 80:20 methanol:tetrahydrofuran as the mobile phase.

### 2.5. Statistical Analyses

Comparison of means between quartiles was performed using the Bonferroni multiple comparison test and the PROC GLM of the statistic software SAS 9.4 (SAS Institute, Cary, NC, USA). The correlation between MDS, lipid-soluble micronutrients, and inflammatory parameters was carried out using the PROC CORR of the statistic software SAS 9.4. The analysis of the response to increasing ingestions of animal or vegetable foods was performed as orthogonal contrast with the PROC GLM of the statistic software SAS 9.4.

## 3. Results

### 3.1. Characteristic of Study Population

A total of 139 volunteers were screened and eligible for the study. After removing the women who had not provided enough samples or data, a total of 80 women with a mean age of 54.9 ± 10.6 were included in the final analysis. The mean BMI value for each quartile was approximately 30, the cut-off point between overweight and obesity. The complete description of patients’ characteristics divided by the quartile of MDS and with reference range values for each biomarker analyzed [31,32,33,34,35] is reported in Table 1.

### 3.2. Association of MDS with Lipid-Soluble Vitamins and Carotenoids

The circulating concentrations of lipid-soluble vitamins such as retinol, tocopherol, and carotenoids are reported in Table 1, and Pearson’s correlation of MDS with these molecules was explored (Table 2). The mean value of MDS was 33.5 ± 4.3. MDS was significantly and positively correlated with lycopene (r 0.274, *p* < 0.05) and β-Carotene plasmatic concentration (r 0.331, *p* < 0.01), while, conversely, it was significantly but negatively associated with retinol (r 0.346, *p* < 0.05). If the data of MDS were disaggregated according to consumption of fruit and vegetables, a positive linear trend with β-carotene levels in blood was detected (*p* < 0.01, Table S1). Similarly, retinol blood concentrations were positively linked to the consumption of foods of animal origin (*p* < 0.05, Table S2) and inversely related to the intake of fruit and vegetables (*p* < 0.001, Table S1).

Moreover, the association between all plant pigments and vitamins was investigated. As expected, β-Carotene, lycopene, β-cryptoxanthin, and tocopherol were positively correlated with MDS (Table 2). Conversely, retinol was negatively associated with β-cryptoxanthin (r −0.231; *p* < 0.05) lycopene (r −0.248, *p* < 0.05), and tocopherol (r −0.405, *p* < 0.001). Finally, vitamin D resulted positively related to lutein-zeaxanthin, β-cryptoxanthin, lycopene, β-carotene, and tocopherol.

### 3.3. Association of MDS with Biomarkers Related to Inflammation

The plasmatic levels of inflammatory biomarkers were also monitored in the cohort of BC survivors (Table 1). As presented in Table 3, a statistically significant inverse association between MDS and FRAP was observed (r −0.256; *p* < 0.05), but none of the other inflammation-related biomarkers analyzed in this study were significantly associated with the score. A statistically significant direct association between ROM and Ceruloplasmin (r 0.736; *p* < 0.01) was found, as expected, as well as an indirect association between PON and haptoglobin (r −0.270; *p* < 0.05) (Table 3). In order to further explore the association between MDS-related biomarkers and inflammation, a correlation of inflammatory biomarkers and lipid-soluble micronutrients was assessed (Table 4).

PON showed a positive relationship with retinol (r 0.454; *p* < 0.001) and a negative relationship with tocopherol (r −0.234, *p* < 0.05). ROMs were also negatively correlated with retinol (r −0.358, *p* < 0.01) and positively with some carotenoids such as lycopene (r 0.323, *p* < 0.01) and tocopherol (r 0.279, *p* < 0.05) (Table 4). No significant relationship was detected between inflammatory markers and vitamin D.

### 3.4. Association of MDS with Cardiometabolic and Anthropometric Parameters

Fasting concentration and indices of glucose and lipid metabolism and BMI are presented in Table 1. Pearson’s correlation of MDS, BMI, and cardiometabolic parameters is presented in Table 5. As expected, an inverse correlation was found between MDS and all the parameters considered, although none of them were significantly associated (*p* ˃ 0.05). Further investigation of the relationship between the parameters showed an expected positive correlation of BMI with fasting glucose (r 0.261, *p* < 0.05), of fasting glucose with fasting insulin (r 0.403, *p* < 0.001) and HOMA-IR (r 0.553, *p* < 0.001), and of TC with LDL-C (r 0.270, *p* < 0.05), HDL-C (r 0.318, *p* < 0.05) and TAG (r 0.806, *p* < 0.001).

## 4. Discussion

This study confirms the hypothesis of an association of MDS with circulating dietary-related biomarkers in a cohort of BC survivors. In particular, we were able to describe that MDS correlated positively with blood levels of β-carotene, lycopene, antioxidant capacity (FRAP), while it was negatively associated with retinol.

Several studies have investigated the association between MD and the circulating concentration of several putatively cancer-protective molecules, which are mostly present in plant-based food groups, such as fruit and vegetables [36,37]. In fact, some biomarkers derived from the diet such as carotenoids, vitamin E, and other bioactive molecules may play an important role in preventing cancer [38]. For this reason, identifying specific biomarkers characteristic of the MD could be a useful and evidence-based approach in order to accurately monitor adherence to this dietary pattern.

The mean values in blood levels of retinol, tocopherol, and vitamin D, found in the group of volunteers included in our study, fell in the normal range for each quartile of MDS. Moreover, blood glucose and TG resulted within the normal range, while total cholesterol and LDL cholesterol were higher than the physiological levels. The women enrolled in this investigation presented haptoglobin levels falling into the normal range, while for ceruloplasmin the mean levels for each quartile were higher than the maximum threshold of physiological value.

This study showed that lycopene, β-carotene, and retinol were significantly correlated with the MDS proposed by Demetriou [24] in this cohort of women treated for BC. As expected, lycopene and β-carotene were positively correlated with MDS, while retinol was inversely associated with MDS. A positive relationship between MDS and carotenoid blood levels was also reported in Fallaize et al.’s paper [39]. Since fruit and vegetables are the main dietary sources of carotenoids [40], the positive relationship of MDS with β-carotene can be easily explained. This result obtained is also useful for identifying a blood marker which allows us to validate adherence to Mediterranean Diet obtained through a food frequency questionnaire.

Moreover, these results can be partially accounted for by the fact that retinol is a vitamin present in meat and derived products, which can reduce adherence to the MD. However, an inverse association between plasma retinol and both carotenoids and tocopherol was found in this study. The mean concentration of β-carotene found in this study (521,8 + 450 µg/L) was higher than the plasmatic concentration previously reported in a similar study, which described a concentration ranging from 171.11 ± 180.2 to 227.1 ± 157.8 µg/L, depending on the stage of the BC [41], and the mean values of 329.3 µg/L described for the women enrolled in the Italian cohorts of the European Prospective Investigation into Cancer and Nutrition [40]. These results were consistent with adherence to the MD, which is based on a high intake of plant foods and low consumption of animal foods.

β-cryptoxanthin plasmatic concentration was lower than the mean values for women reported in the EPIC-based study of Al-Delaimy et al. (2004) [40] (0.29 vs. 0.45 µM/L), but very similar to the concentration determined in another EPIC-based study [38], where vitamins and plant pigments were determined in BC affected women. All the results obtained in our study fell within the range of physiological variability observed in the available literature.

Regarding two parameters related to inflammation, haptoglobin, and ceruloplasmin, only for the latter were there observed mean values higher than the physiological threshold. This could be explained by the increased level of this molecule in women using the contraceptive pill [42]. Since in our cohort the menstrual cycle was blocked also in premenopausal women, higher levels of ceruloplasmin may be due to anti-estrogen treatment. Furthermore, the relationship between inflammatory parameters and MDS was explored. Only antioxidant capacity was significantly and negatively correlated with MDS, whereas none of the analyzed biomarkers were associated with it. However, we do not have an explanation for this result. In contrast with our result, a previous study described an amelioration of serum antioxidant capacity in BC survivors following the MD [18]. The inverse relationship between retinol and ROMs may be explained by the antioxidant properties of vitamin A, while the positive correlation of ROMs—inflammation and oxidative stress markers—with lycopene and tocopherol is unexpected and hardly justifiable. Haptoglobin, another molecule whose levels increase during inflammation, was not affected by the blood concentration of plant pigments in this investigation. Both these findings are in accordance with D’Odorico et al. [43], who reported the absence of a relationship between retinol, tocopherol, and carotenoids with the C-reactive protein, a well-known inflammation marker. Nevertheless, both C-reactive protein and haptoglobin are related to the acute effects of inflammation and much less to the chronic consequences of this process. Conversely, ceruloplasmin seems to be a biomarker of the chronic consequences of inflammation as also demonstrated by the marked correlation with ROMs. PON blood level is the result of a complex interaction between nutrient intake (vitamins, antioxidants from plant foods, dietary fat) and genetic background [44]. Therefore, the relationship between PON and the vitamins of plant molecules is not easily predictable. Diets rich in plant food have caused both an increase [45] and a reduction in the concentration of PON [46].

The positive relationship of PON with retinol and tocopherols has been frequently observed [47] and is due to the anti-oxidative action of these vitamins, which, counteracting oxidative stress, reduce the need to synthesize PON. In our study, only retinol confirmed this pattern, while vitamin E resulted inversely related to PON levels in the blood. This unexpected result could be due to the low level of inflammation in our volunteers (haptoglobin concentration was within normal range).

It is also important to note that we did not consider other bioactive molecules, such as polyphenols, which are well known to exert antioxidant activity in humans. Moreover, inflammation may also be dependent on other lifestyle factors. To date, a large body of evidence supports the role of antioxidant biomarkers such as vitamin A, vitamin E (α-tocopherol), and vitamin C in the prevention of BC and in BC survival, although there are still conflicting results. In previous studies, women with higher levels of vitamin C, α-carotene, β-carotene, or cryptoxanthin appeared to have a lower risk of BC than women with low levels of these antioxidants, while retinol was positively correlated with BC risk in relation to ER2/progesterone receptor–negative tumors [38]. Another systematic review and meta-analysis provided limited evidence regarding pre-diagnosis dietary intake of β-carotene and the overall survival of women with BC [48], while a recent systematic review supported the role of β-carotene in BC prevention [49]. A recent study suggested that pre-diagnosis concentrations of vitamin E were moderately—strongly associated with ER-BC risk after analysis with a metabolomic approach [50]. A 20-year Nurses’ Health Study carried out on women indicated that carotenoids were associated with a low risk of BC onset [51].

Concerning the correlation of MDS and the lipid-soluble vitamins analysed with the cardiometabolic parameters analyzed, unexpectedly, we did not find a significant association. Different results were obtained by Skouroliakou and colleagues who found an amelioration of serum antioxidant and cardiometabolic parameters in BC survivors, which was associated with higher vitamin C, polyunsaturated fatty acids, vitamin A and a-tocopherol levels than in the control non-MD treated group [18]. The association between MDS and cardiometabolic parameters and BMI, which are indicators of overall health status and possibly implicated in the recurrence of BC or in the onset of other comorbidities or secondary cancers, was investigated. As expected, all the parameters tended to be positively or negatively correlated with MDS, although no statistical significance was found for any of them. The improvement of cardiometabolic parameters is regarded as positive, especially for patients who are more likely to develop chronic diseases subsequent to the primary cancer.

Several reports have investigated the association between specific food groups and the risk of BC. A meta-analysis of prospective studies concluded that a high intake of fruit, and fruit and vegetables together, but not only vegetables, is associated with a slight reduction of BC risk [37], while no significant associations between fruit and vegetable intake and BC prognosis were found [52]. A very recent study on the EPIC cohort demonstrated a positive association of alcohol consumption and suggested an inverse association of dietary fiber and possibly fruit intake with the risk of BC [53]. In our study, the correlation between specific food groups with serum molecules was investigated and a significant effect was found, but since these data are only related to the baseline status of the women enrolled, no conclusions can be drawn on the long-term effects on the recurrence of BC.

## 5. Conclusions

This study was focused on the evaluation of MDS with dietary-related biomarkers in a cohort of breast cancer survivors. The results indicated that MDS was associated with some circulating lipid-soluble antioxidant vitamins, thus allowing us to evaluate adherence to the Mediterranean Diet in a more objective way than by administering food frequency questionnaires. Nevertheless, MDS did not correlate with inflammation-related biomarkers. This study will add knowledge to the literature on the identification of circulating blood molecules in BC survivors following the MD. Future studies will better explore the presence of other bioactive compounds and, possibly, a comprehensive metabolomic approach will allow us to better investigate the footprint of the MD and other dietary patterns in BC survivors. A future stage of the project will be focused on exploring the effect of the MD on BC relapse, mortality rate, anthropometric and metabolic-related blood parameters, and the overall quality of life of these patients in a medium-long term investigation.

## Figures and Tables

**Table 1:** Characteristics of enrolled patients.

		MDS Quartiles				
	Reference Range Values	Q1	Q2	Q3	Q4	RMSE
MDS	-	28.05	32.88	35.53	38.00	131.17
BMI, kg/m^2^	18.5–24.9 [31]	30.8	29.7	30.2	29.3	8.35
Glucose, mg/dL	70–100 [32]	91.2	97.4	99.9	85.3	17.05
Insulin, µU/mL	-	12.8	13.2	8.52	8.7	8.53
HOMA-IR	-	3.06	3.32	2.26	1.92	2.44
TC, mg/dL	<200 [33]	230.0	228.1	206.5	239.1	61.07
LDL-C, mg/dL	<100 [33]	148.8	140.0	135.2	132.4	37.59
HDL-C, mg/dL	>40 [33]	59.1	67.2	52.1	60.3	15.56
TAG, mg/dL	<150 [33]	135.5	130.8	122.4	143.0	64.53
Vitamin D ng/mL	20–150 [34]	17.7	16.8	19.0	22.4	9.54
Lutein-Zeaxanthin, mg/L	-	0.30	0.30	0.30	0.33	0.15
β-Cryptoxanthin, µg/L	-	136.2	134.8	175.7	191.3	126.8
Lycopene, µg/L	-	0.41	0.59	0.91	0.75	0.64
Retinol, µg/dL	20–86 [34]	66.9	58.0	49.8	50.2	22.45
Tocopherol, µg/mL	5–20 [34]	12.7	14.4	13.4	13.9	4.63
β-Carotene, µg/L	-	320.0 ^a^	438.7 ^ab^	679.1 ^ab^	684.6 ^b^	429.01
Ceruloplasmin, µmol/L	1.3–3.4 [35]	3.42	3.75	3.55	3.83	1.05
Haptoglobin, g/L	0.9–2.3 [34]	1.06	1.10	1.10	1.15	0.27
Paraoxonase, U/mL	-	182.0	180.0	171.3	171.5	32.41
ROM, mg H_2_O_2_/dL	-	44.3	47.9	47.4	49.8	9.59
SHp, µmol/L	-	371.1	346.2	328.6	384.0	121.4
FRAP, µmol/L	-	528.9	503.6	503.7	471.8	116.4

BMI: Body Mass Index; HDL-C: HDL-cholesterol HOMA-IR: Homeostatic Model Assessment of Insulin Resistance; LDL-C: LDL-cholesterol; LZ: Lutein + Zeaxanthin; MDS: Mediterranean Diet Score; RMSE: root mean square error; ROMs: reactive oxygen metabolites; SHp: thiol group; TAG: triglycerides; TC: Total cholesterol. ^a,b^ Different letters indicate statistical significance (*p* < 0.05).

**Table 2:** Correlation (r) of MDS with lipid-soluble micronutrients.

	MDS	LZ	β-Cryptoxanthin	Lycopene	Retinol	Tocopherol	β-Carotene	Vitamin D
MDS	1.000	0.091	0.190	0.274 *	−0.346 *	0.054	0.331 **	0.152
LZ		1.000	0.193	0.199	0.261 *	−0.032	0.188	0.249 *
β-Cryptoxanthin			1.000	0.476 ***	−0.231 *	0.369 **	0.484 ***	0.324 **
Lycopene				1.000	−0.248 *	0.282 *	0.466 ***	0.338 **
Retinol					1.000	−0.405 ***	−0.224 *	−0.147
Tocopherol						1.000	0.270 *	0.269 *
β-Carotene							1.000	0.359 ***
Vitamin D								1.000

* *p* < 0.05; ** *p* < 0.01; *** *p* < 0.001; LZ: Lutein + Zeaxanthin.

**Table 3:** Correlation (r) between Mediterranean Score (MDS) and markers of inflammation.

	MDS	Ceruloplasmin	Haptoglobin	Paraoxonase	ROMs	SHp	FRAP
MDS	1.000	0.110	0.114	−0.096	0.199	0.012	−0.256 *
Ceruloplasmin		1.000	−0.015	−0.022	0.736 **	−0.024	0.1503
Haptoglobin			1.000	−0.270 *	0.090	0.076	−0.066
Paraoxonase				1.000	−0.160	0.170	−0.116
ROMs					1.000	0.024	0.021
SHp						1.000	0.114
FRAP							1.000

* *p* < 0.05; ** *p* < 0.01; FRAP: frap ferric reducing antioxidant power; LZ: Lutein + Zeaxanthin; MDS: Mediterranean Diet Score; ROMs: reactive oxygen metabolites; SHp: thiol group.

**Table 4:** Correlation (r) between lipid-soluble micronutrients and markers of inflammation.

	LZ	β-Cryptoxanthin	Lycopene	Retinol	Tocopherol	β-Carotene	Vitamin D
Ceruloplasmin	−0.170	−0.116	0.144	−0.044	−0.080	−0.101	−0.138
Haptoglobin	−0.158	0.015	0.039	−0.084	0.029	0.056	0.037
Paraoxonase	0.171	0.018	−0.018	0.454 ***	−0.234 *	−0.118	−0.155
ROMs	−0.181	0.052	0.323 **	−0.358 **	0.279 *	0.028	0.083
SHp	−0.010	0.058	0.1445	−0.116	−0.039	0.042	0.002
FRAP	0.033	−0.031	0.063	0.196	0.052	−0.066	−0.092

* *p* < 0.05; ** *p* < 0.01; *** *p* < 0.001; FRAP: frap ferric reducing antioxidant power; LZ: Lutein + Zeaxanthin; MDS: Mediterranean Diet Score; ROMs: reactive oxygen metabolites; SHp: thiol group.

**Table 5:** Associations MDS and cardiometabolic and anthropometric variables.

	MDS	BMI	Glucose	Insulin	HOMA-IR	TC	LDL-C	HDL-C	TAG
MDS	1.000	−0.110	−0.216	−0.199	−0.176	−0.024	−0.192	−0.018	0.109
BMI		1.000	0.261 *	0.206	0.196	0.070	0.168	−0.171	0.025
Glucose			1.000	0.403 ***	0.553 ***	−0.002	0.094	−0.147	−0.064
Insulin				1.000	0.974 ***	−0.078	−0.088	−0.306 **	0.0364
HOMA-IR					1.000	−0.078	−0.088	−0.305 **	0.036
TC						1.000	0.270*	0.318 **	0.806 ***
LDL-C							1.000	−0.091	−0.278 *
HDL-C								1.000	0.128
TAG									1.000

BMI: Body Mass Index; HDL-C: HDL-cholesterol; HOMA-IR: Homeostatic Model Assessment of Insulin Resistance; LDL-C: LDL-cholesterol; MDS: Mediterranean Diet Score; TAG: triglycerides; TC: Total cholesterol. * *p* < 0.05; ** *p* < 0.01; *** *p* < 0.001.

## Supplementary Materials

The following are available online at https://www.mdpi.com/article/10.3390/nu13103482/s1, Table S1: Blood levels of metabolic parameters, plant pigments and vitamin as a function of fruit and vegetable intake, Table S2: Blood levels of metabolic parameters, plant pigments and vitamin as a function of animal foods intake.
