# Supplementary material for: Mediterranean Diet Affects Blood Circulating Lipid-Soluble Micronutrients and Inflammatory Biomarkers in a Cohort of Breast Cancer Survivors: Results from the SETA Study
Source: Nutrients. 2021 Sep 30;13(10):3482. doi: 10.3390/nu13103482 (PMC8539583; doi:10.3390/nu13103482)
Supplement: Supplementary file 1 [file nutrients-13-03482-s001.zip › nutrients-1391090-supplementary.pdf]

Table S1. Blood levels of metabolic parameters, plant pigments and vitamin as a function of fruit and vegetable intake

	Quartile of fruit and vegetables intake				RMSE	P for linear response
	Quartile 1	Quartile 2	Quartile 3	Quartile 4		
Metabolic parameters						
Ceruloplasmin, µmol/L	3.62	4.07	3.49	3.33	1.10	0.2964
Haptoglobin, g/L	1.04	1.07	1.21	1.12	0.29	0.2571
GOT, U/L	30.10	24.48	18.17	21.32	20.1	0.1440
GPT, U/L	17.3	13.4	8.2	9.6	16.2	0.1187
GGT, U/L	49.0	36.3	13.1	23.8	63.2	0.1643
Bilirubin, µmol/L	7.36	6.86	6.67	5.45	3.51	0.1368
Paraoxonase, U/mL	195.96	173.85	174.44	168.04	31.90	0.0178
ROM, mg H_2O_2/dL	43.28	50.80	46.33	45.95	9.65	0.6195
Thiolic groups, µmol/L	335.5	338.8	299.6	357.6	81.1	0.7630
FRAP, µmol/L	513.3	521.1	488.1	446.3	118.2	0.0917
Plant pigments						
Lutein+zeaxanthin, mg/L	0.32	0.35	0.27	0.27	0.15	0.1747
Β-Cryptoxanthin, µg/L	143.6	100.7	190.6	183.7	129.9	0.2001
Lycopene, µg/L	0.47	0.57	0.64	0.32	0.54	0.5747
Vitamins and pro-vitamins						
Retinol, µg/dL	75.71	62.93	49.20	47.58	22.1	0.0002
Tocopherol, µg/mL	11.17	11.55	12.98	16.88	3.54	<0.0001
β-carotene, µg/L	29.27	40.52	48.30	64.84	36.5	0.0067

ALA: Alanine Aminotransferase; AST: Aspartate aminotransferase; FRAP: frap ferric reducing antioxidant power; GGT: Gamma-glutamyl transpeptidase; RMSE = Root Mean Square Error; SHp: thiol group

Table S2. Blood levels of metabolic parameters, plant pigments and vitamin as a function of animal foods intake

	Quartile of animal foods intake				RMSE	P for linear response
	Quartile 1	Quartile 2	Quartile 3	Quartile 4		
Metabolic parameters						
Ceruloplasmin, μmol/L	3.65	3.67	3.82	3.90	1.12	0.5031
Haptoglobin, g/L	1.09	1.08	1.10	1.13	0.30	0.7913
AST, U/L	17.7	24.0	23.3	37.3	19.4	0.0195
ALA, U/L	8.2	11.7	10.9	24.2	15.6	0.0247
GGT, U/L	24.3	23.4	37.9	55.6	63.4	0.2040
Bilirubin, μmol/L	6.20	7.11	5.93	7.36	3.54	0.5844
Paraoxonase, U/mL	162.5	183.5	186.3	192.2	31.6	0.0066
ROM, mg H_2O_2/dL	47.9	45.1	45.8	47.8	10.0	0.7968
SHp, μmol/L	315.6	353.9	338.7	342.8	81.9	0.3283
FRAP, μmol/L	461.3	516.7	510.9	500.2	119.7	0.2564
Plant pigments						
Lutein+zeaxanthin, mg/L	0.24	0.34	0.31	0.39	0.15	0.0118
Β-Cryptoxanthin, μg/L	161.1	201.4	167.4	130.1	130.3	0.5194
Lycopene, μg/L	0.22	0.50	0.79	0.60	0.51	0.0074
Vitamins and pro-vitamins						
Retinol, μg/dL	47.7	64.2	65.6	70.6	23.2	0.0048
Tocopherol, μg/mL	14.7	13.6	11.6	10.7	3.9	0.0040
β-carotene, μg/L	48.1	47.5	50.8	28.9	38.2	0.3539

ALA: Alanine Aminotransferase; AST: Aspartate aminotransferase; FRAP: frap ferric reducing antioxidant power; GGT: Gamma-glutamyl transpeptidase; RMSE = Root Mean Square Error; SHp: thiol group
